# Sulfonic‐Pendent Vinylene‐Linked Covalent Organic Frameworks Enabling Benchmark Potential in Advanced Energy

**DOI:** 10.1002/advs.202300408

**Published:** 2023-03-01

**Authors:** Ying Xu, Zhiwu Yu, Qingyun Zhang, Feng Luo

**Affiliations:** ^1^ School of Chemistry Biology and Materials Science East China University of Technology Nanchang 330013 China; ^2^ High Magnetic Field Laboratory Chinese Academy of Sciences Hefei Anhui 230031 China

**Keywords:** knoevenagel condensation, proton conductivity, sulfonic group, sensing, UO_2_^2+^ capture, vinylene‐linked COFs

## Abstract

Both proton exchange membrane fuel cells and uranium‐based nuclear techniques represent two green and advanced energies. However, both of them still face some intractable scientific and industrial problems. For the former, established proton‐conduction materials always suffer one or another defect such as low proton conductivity, high activation energy, bad durability, or just small‐scale product; while for the later, there still lacks available adsorbent to selectively recover of UO_2_^2+^ from concentrated nitric acid (>1 M) during the spent fuel reprocessing due to the deactivation of the adsorption site or the decomposition of adsorbent under such rigorous conditions. It is found that the above two issues can be well solved by the construction of sulfonic‐pendent vinylene‐linked covalent organic frameworks (COFs), since these COFs contain abundant sulfonic units for both intrinsic proton conduction and UO_2_^2+^ capture through strong coordination fixation and vinylene linkage that enhances the stability up to 12 M nitric acid (one of the best materials surviving in 12 M HNO_3_).

## Graphical abstract

Large‐scale synthesis of methylsulfonic‐derived vinylene‐linked covalent organic frameworks is for the first time achieved through using an HSO_3_‐activated approach. The resultant covalent organic frameworks afford not only ultrahigh chemical stability, but also benchmark potential in both proton exchange membrane fuel cells and uranium‐based nuclear energy.

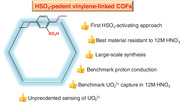

## 1 Introduction

Proton exchange membrane fuel cells (PEMFCs) as one of the most promising candidates among fuel cells are now attracting explosive attention due to their outstanding advantages like that of mild operating temperature, high energy conversion efficiency, and high power density.^[^ ^1^, ^2^, ^3^ ^]^ For PEMFCs, a crucial component is the solid‐state proton conductors. Generally, commercial Nafion membranes showing proton conductivities of 10^−1^‐10^−2^ S cm^−1^ under 60—80 °C and 98% relative humidity (RH) was used. However, its high manufacturing cost plus prolonged start‐up time derived from high activation energy (>0.1 eV) inherently limits its wide‐spread use.^[^ ^4^ ^]^ Similarly, always one or another disadvantage was observed for established solid‐state proton conduction materials including metal oxides, mesoporous silica, metal‐organic frameworks (MOFs),^[^ ^5^ ^]^ covalent organic frameworks (COFs),^[^ ^3^ ^]^ and hydrogen‐bonded frameworks.^[^ ^6^ ^]^ For example, IL‐COF‐SO_3_H@SNF^[^ ^7^ ^]^ and H_3_PO_4_@TPB‐DMeTP‐COF^[^ ^8^ ^]^ displayed high proton conductivity, but they suffered from low durability. H_3_PO_4_@NKCOF‐10^[^ ^9^ ^]^ afforded high proton conductivity, low activation energy, and large‐scale manufacture, but its low durability (just 24 h) seriously restricts its practical industrial application. The low durability for the above‐reported materials is mainly due to the gradual loss of extrinsic proton‐conduction source such as H_3_PO_4_ in most cases. NUS‐10(R)^[^ ^10^ ^]^ containing ‐HSO_3_ intrinsic proton conduction showed moderate proton conductivity and excellent durability, but suffered relatively high activation energy and small‐scale manufacture. Two new ‐SO_3_ MOFs were reported to show good proton conduction, where BUT‐8(Cr)A^[^ ^11^ ^]^ afforded high proton conductivity and low activation energy, but weak durability, and MFM‐300(Cr)·SO_4_(H_3_O)_2_^[^ ^12^ ^]^ enabled low activation energy and good durability, but low proton conductivity. In this regard, there urgently needs to seek for new proton‐conduction material with high proton conductivity, low activation energy, excellent durability, and large‐scale manufacture together.

On the other hand, uranium‐based nuclear energy, as a clean and advanced energy, is attracting wide attention. However, from the viewpoint of sustainable development path, there emerged an urgent concern about the recovery of uranium from spent fuel which contains >90% unconsumed uranium.^[^ ^13^ ^]^ Generally speaking, to recovery of uranium from the spent fuel, we must resolve several issues.^[^ ^14^ ^]^ First, the employed adsorbents must undergo such extremely rigorous environments such as 12 M HNO_3_ (generally used to dissolve spent fuel) and strong irradiation (>100 kGy). However, there is still seriously lacks of adsorbents available from commonly established porous materials such as crystalline metal‐organic frameworks and COFs, and amorphous polymers, since only limited case can survive in such extremely rigorous environments.^[^ ^15^, ^16^ ^]^ Furthermore, given that the employed adsorbent can survive from such condition, however, validity becomes to be another concern, because the adsorption site for uranium would be invalid under 12 M HNO_3_, due to serious protonation effect. In this regard, future efforts should be made on designing strong adsorption site that could be valid under 12 M HNO_3_. In addition to tolerance and validity of materials, selectivity represents another key factor, since the spent fuel contains more than 30 other metal elements. Moreover, we also should consider the synthesis of adsorbent from the viewpoint of industrial application, such as low‐cost, green, and large‐scale synthetic method is a highly desirable one.

**Scheme 1:**
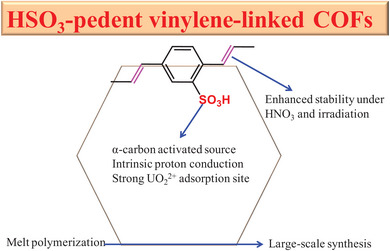
A proposed draft of construction of HSO_3_‐pendent vinylene‐linked COFs for solving the above‐mentioned two problems in advanced energy of PEMFCs and uranium‐based nuclear technique.

To solve the above two issues, construction of sulfonic‐pendent materials could be the best choice (**Scheme** **1**). Previous reports have revealed the key role of sulfonic units in both intrinsic proton conduction^[^ ^10^ ^]^ and UO_2_^2+^ capture.^[^ ^17^ ^]^ However, these established sulfonic‐pendent materials still suffered small‐scale synthesis and low stability under concentrated nitric acid,^[^ ^17^ ^]^ thus seriously restricting their practical industrial applications in the above‐mentioned two advanced energies. To solve the stability problem, a large number of literature studies show that the best choice is to construction of sulfonic‐pendent vinylene‐linked COFs. As we know, Knoevenagel condensation can be employed to prepare vinylene‐linked COFs when using *α*‐carbon activated monomers such as cyano group and pyridyl nitrogen.^[^ ^18^ ^]^ It is quite interesting to note that the sulfonic group (‐HSO_3_) holds comparable electron‐withdrawing ability as observed in cyano group and pyridyl nitrogen, capable of promising activating unit for Knoevenagel condensation and succedent preparation of vinylene‐linked COFs. But to the best of our knowledge, there still lack of example of vinylene‐linked COF constructed from *α*‐carbon activated sulfonic‐containing monomer. On the other hand, to solve the large‐scale generation problem, the recently developed melt polymerization method could be the best alternative.^[^ ^19^ ^]^

To this end, two new HSO_3_‐pendent vinylene‐linked COFs were synthesized using TBS (2,4,6‐trimethylbenzenesulfonic acid) and DBS (2,5‐dimethylbenzenesulfonic acid) as the *α*‐carbon activated monomer through melt polymerization. The obtained materials showed moderate crystallinity and porosity, but ultrahigh chemical stability under 12 M HNO_3_ and 200 kGy irradiation. Sulfonic unit is found to play a key role not only in the synthesis of vinylene‐linked COFs through Knoevenagel condensation, but also in their significant applications such as proton conduction, UO_2_^2+^ capture, and sensing.

## 2 Results and Discussion

**Figure 1:**
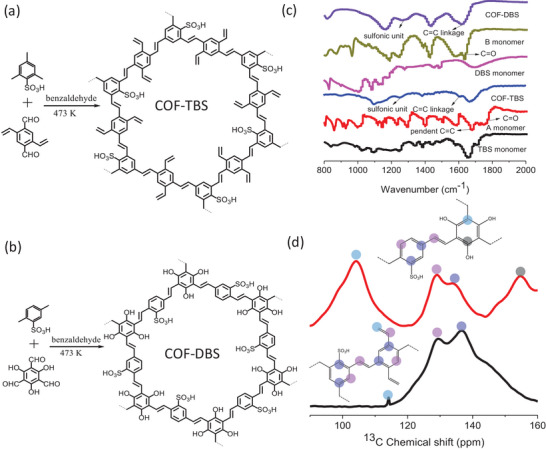
a,b) The schematic description of the synthesis of COF‐TBS and COF‐DBS. c) IR characterizations of COF‐TBS and COF‐DBS, referring to corresponding monomers. d) CPMAS‐ssNMR characterizations of COF‐TBS and COF‐DBS with the assignment of all peaks.

COF‐TBS and COF‐DBS were synthesized from Knoevenagel condensation between TBS and 2,5‐divinylterephthalaldehyde (A) or DBS and 2,4,6‐trihydroxybenzene‐1,3,5‐tricarbaldehyde (B) (**Figure** **1**a,b). Benzoic anhydride was used for melt polymerization with reaction temperature of 200 °C. The detail of synthesis was included in Supporting Information and gram‐scale synthesis can be achieved for one batch reaction. The corresponding model compounds were prepared using TBS and benzaldehyde or DBS and 2‐hydroxybenzaldehyde, respectively, and confirmed by mass spectrometry (HR‐MS) with a yield of >96% (Figure S1–S2, Supporting Information).

The chemical structures of these COFs were initially characterized by Fourier transform infrared (IR) and ^13^C cross polarization magic angle spinning (CPMAS) solid‐state nuclear magnetic resonance (CPMAS‐ssNMR) spectroscopy. In IR spectra (Figure 1c), the observation of new peak at 1606 cm^−1^ in COF‐TBS and 1619 cm^−1^ in COF‐DBS were attributed to *trans*‐C=C linkage, and accompanied was the absence of C=O stretching vibrations.^[^ ^18^, ^19^ ^]^ The presence of sulfonic group was attested by peak at 1257 cm^−1^ in COF‐TBS and 1271 cm^−1^ in COF‐DBS,^[^ ^17^ ^]^ where the blue‐shift in COF‐TBS is due to the bigger steric hindrance for the sulfonic group in TBS fragment, relative to COF‐DBS. In the CPMAS‐ssNMR spectra (Figure 1d), the signal at 114 ppm in COF‐TBS was assigned to the terminal carbon atoms of free‐standing vinyl group from A monomer, while peak at 105 ppm in COF‐DBS belonged to the carbon atoms from B connecting to the resultant trans‐C=C linkage. Other peaks were also reasonably assigned and shown in Figure 1d. All these results essentially verified the structure of HSO_3_‐pendent vinylene‐linked COFs.

**Figure 2:**
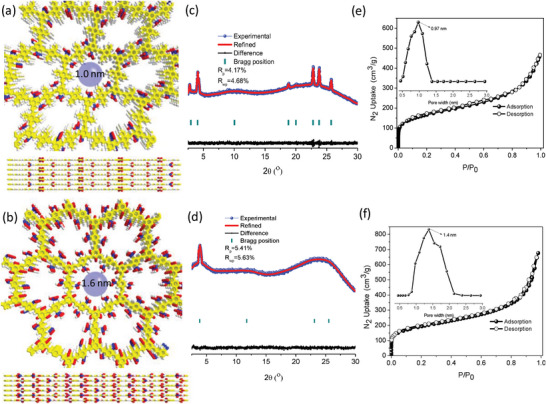
a,b) View of the structures of COF‐TBS and COF‐DBS. c,d) PXRD patterns of COF‐TBS and COF‐DBS with a comparison among the experimental profiles (blue), Pawley refined profiles (red), the Bragg positions (green), and the refinement differences (black). e,f) N_2_ adsorption isotherms at 77 K for COF‐TBS and COF‐DBS with the inset of pore distribution.

Scanning electron microscopy images revealed the particulate morphology of these vinylene‐linked COFs; and accompanied was uniform distribution of C, O, and S elements in the energy dispersive spectrometry (EDS) (Figures S3 and S4, Supporting Information). Powder X‐ray diffraction (PXRD) revealed moderate crystallinity of COF‐TBS and COF‐DBS, and their structures were obtained through pawley refinement of PXRD data (**Figure** **2**a‐d). For COF‐TBS, it showed monoclinic crystal system with *Pm* space group and cell parameters of a = 24.60 Å, b = 6.82 Å, c = 24.22 Å, *α* = *γ* = 90˚, and *β* = 126.16˚ for COF‐TBS (Figure 2a,c). For COF‐DBS, hexagonal crystal system with *P6_3_/m* space group was observed with cell parameters of a = b = 23.53 Å, b = 6.82 Å, *α* = *β* = 90˚, and *γ* = 120˚(Figure 2b,d). Both of them showed the eclipsed (AB) stacking model. The content of sulfonic units inside COF‐TBS is below than that in COF‐DBS, because of their connecting fashion. Moreover, irregular or regular 1D channel with a pore size of 1.0 nm or 1.6 nm was observed in COF‐TBS and COF‐DBS, respectively. Nitrogen sorption at 77 K revealed type I reversible isotherms (Figure 2e,f), indicative of microporosity of them. Their Brunauer—Emmett–Teller (BET) surface area is 584 and 672 m^2^ g^−1^, respectively, with corresponding pore volume of 0.33 cm^3^ g^−1^ and 0.38 cm^3^ g^−1^. Narrow pore size distribution was observed with the maxima at 0.97 and 1.4 nm, respectively, well in line with the predicted pore diameter from structural analysis.

**Figure 3:**
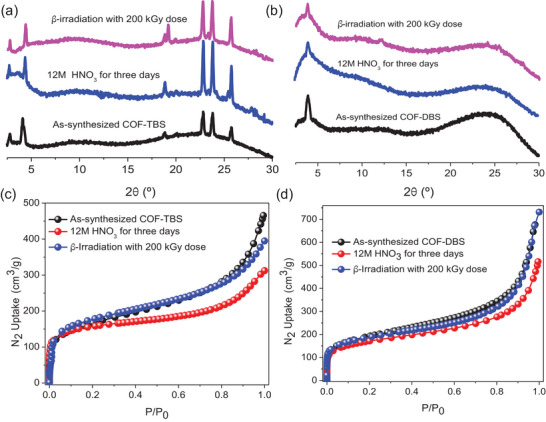
a,b) A comparison of PXRD patterns among as‐synthesized samples of COF‐TBS and COF‐DBS, and the samples after soaking in 12 M HNO_3_ for three days, and the samples after *β*‐irradiation with 200 kGy dose. c,d) A comparison of N_2_ adsorption isotherms at 77 K among as‐synthesized samples of COF‐TBS and COF‐DBS, and the samples after soaking in 12 M HNO_3_ for three days, and the samples after *β*‐irradiation with 200 kGy dose.

For practical applications, especially in the spent fuel reprocessing, the stability of materials presents a key concern. In the literature, it was found that COFs often afforded outstanding chemical stability such as in water, acid, and alkali. However, few COF could survive from concentrated nitric acid, because of its strong acidity and oxidability. To evaluate the HNO_3_ stability of these COFs, the samples of them were first soaked in HNO_3_ (12 M) for one week, and then PXRD (**Figure** **3**a,b) characterization was carried out, and no detectable structural change was observed even under these rigorous conditions, indicative of ultrahigh HNO_3_ stability of these HSO_3_‐pendent vinylene‐linked COFs. To further confirm this, N_2_ adsorption was tested and the results were shown in Figure 3c,d, giving comparable N_2_ adsorption isotherms for samples after soaking in HNO_3_ (12 M). Moreover, COF‐TBS and COF‐DBS also afforded stability after *β*‐irradiation with 200 kGy doses (Figure 3a–d). All the results strongly support their superior application in the spent fuel reprocessing.

**Figure 4:**
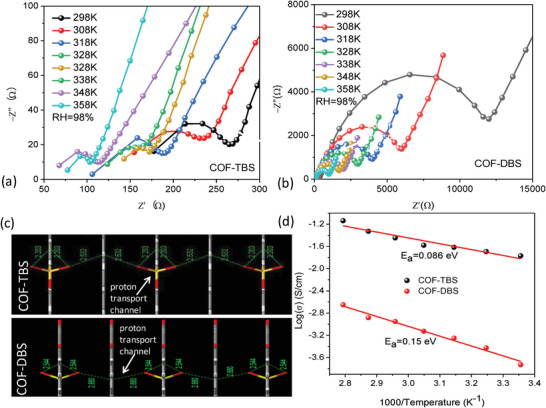
a,b) Nyquist plots for COF‐TBS and COF‐DBS at 298–358 K under 97% RH. c) Proposed proton transport channel from structural data for COF‐TBS and COF‐DBS. (d) Arrhenius plots for COF‐TBS and COF‐DBS.

Confinement of sulfonic unit in materials has been attested to be one of the best effective methods to boost the intrinsic proton conduction ability.^[^ ^10^, ^11^, ^12^ ^]^ However, material containing free‐standing sulfonic units showing high chemical stability remains scarce.^[^ ^17^ ^]^ Accordingly, the exceptional chemical stability observed in the current HSO_3_‐pendent vinylene‐linked COFs promoted us to investigate their proton conduction ability. Moreover, one outstanding advantage of melt polymerization synthesis is the formation of hard block object with the maintenance of shape even after soaking in water, common solvents (such as DMF, CH_3_OH, C_2_H_5_OH, THF), and 12 M HNO_3_ solution (Figure S5, Supporting Information). This allowed for determining their proton conduction performance directly without making tablet, since making tablet under an incorrect fashion would largely reduce its proton conduction performance.^[39]^ The proton conductivity of COF‐TBS and COF‐DBS was analyzed by AC impedance spectroscopy at various temperatures under 98% relative humidity (RH). The proton conductivity of COF‐TBS was measured to be 1.7 × 10^−2^ S cm^−1^ at 298 K (**Figure** **4**a), belonging to the range of superprotonic conductivity, exceeding most reported proton‐conducting materials,^[39]^ and comparable to that of the best‐performing materials reported to date (Table S1, Supporting Information), such as commercial Nafion (5 × 10^−2^ S cm^−1^),^[^ ^2^ ^]^ MOFs of MFM‐300(Cr)·SO_4_(H_3_O)_2_ (1.26 × 10^−2^ S cm^−1^),^[^ ^12^ ^]^ BUT‐8(Cr)A (7.61 × 10^−2^ S cm^−1^),^[^ ^11^ ^]^ MIL‐101‐SO_3_H (1.1 × 10^−2^ S cm^−1^),^[^ ^11^ ^]^ and COFs of NUS‐9 (R) (1.24 × 10^−2^ S cm^−1^),^[^ ^10^ ^]^ H_3_PO_4_@NKCOF‐10 (6.97 × 10^−2^ S cm^−1^)^[^ ^9^ ^]^. More impressively, the value in COF‐TBS should represent the highest one among all reported COFs with the proton conduction achieved through intrinsic property. By contrast, COF‐DBS afforded proton conductivity of 1.87 × 10^−4^ S cm^−1^ under the same condition (Figure 4b), which is two orders of magnitude lower than that in COF‐TBS. This is mainly because COF‐TBS enables more effective proton transport channel through hydrogen bonds than that observed in COF‐DBS (Figure 4c), although both of them contains free‐standing ‐HSO_3_ units as intrinsic source for proton conduction.

**Figure 5:**
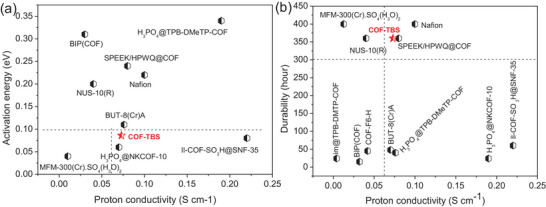
A comparison of proton conduction performance between reported outstanding materials and our case in proton conductivity versus activation energy a) plus proton conductivity versus durability b). For both Nafion and MFM‐300(Cr)·SO_4_(H_3_O)_2_, their real durability is more than 400 h, herein, only 400 h was used to give a clear comparison.

The proton conductivity for COF‐TBS and COF‐DBS was further examined at elevated temperatures of up to 358 K, and the results were shown in Figure 4. The proton conductivity ability increasing along with increasing temperature was observed for both of them, giving high proton conductivity of 7.28 × 10^−2^ S cm^−1^ for COF‐TBS and 2.24 × 10^−3^ S cm^−1^ for COF‐DBS (Figure 4a,b), respectively. For COF‐TBS, the proton conductivity was found to be stable among 298–358 K and just a little dependent on temperature, resulting in an ultralow activation energy of 0.086 eV (Figure 4d), lower than almost all reported materials for such use (Table S1, Supporting Information), but slightly higher than MFM‐300(Cr)·SO_4_(H_3_O)_2_ (0.04 eV)^[^ ^12^ ^]^, H_3_PO_4_@NKCOF‐10 (0.06 eV),^[^ ^9^ ^]^ and H_3_PO_4_@NKCOF‐4 (0.08 eV).^[^ ^9^ ^]^ Notably, if excluding the extrinsic proton conductivity, the activation energy in COF‐TBS should represent the lowest one in all reported materials (Table S1, Supporting Information). By contrast, temperature‐dependent proton conductivity was observed in COF‐DBS, giving relatively high activation energy of 0.15 eV (Figure 4d). Based on their activation energy value, Grotthuss mechanism was suggested for their excellent proton transport.^[^ ^3^ ^]^ Furthermore, durability represents another crucial index for practical application.^[^ ^3^ ^]^ However, the major limitation is still on their durability for most established proton conduction materials, mainly due to the inherent low chemical stability of materials or the leakage of extrinsic proton conductivity.^[^ ^3^ ^]^ Remarkably, it was found that the high proton conductivity of COF‐TBS can be retained upon continuous run over 15 days (Figure S6, Supporting Information), suggesting ultrahigh durability,^[^ ^3^, ^7^, ^8^, ^9^ ^]^ which is mainly due to the exceptional stability of vinylene‐linked framework, the intrinsic ‐HSO_3_ proton conductivity, and the determination fashion (without making tablet). For example, the benchmark materils of Il‐COF‐SO_3_H@SNF‐35^[^ ^7^ ^]^ and H_3_PO_4_@NKCOF‐10^[^ ^9^ ^]^ just showed durability 48 and 24 h, respectively. A comprehensive consideration including in proton conduction ability, activation energy, and durability suggests our case of COF‐TBS being the best material among all established proton conduction materials (**Figure** **5**, Table S1, Supporting Information).^[^ ^3^ ^]^ For example, materials with proton conductivity (>0.05 S cm^−1^) and activation energy (<0.1 eV) were just observed in H_3_PO_4_@NKCOF‐10, Il‐COF‐SO_3_H@SNF‐35, and our case of COF‐TBS, while materials with proton conductivity (>0.05 S cm^−1^) and durability (>300 h) was just observed in SPEEK/HPWQ@COF, Nafion, and our case of COF‐TBS. The results reveal that only our case of COF‐TBS can meet all the criterion. More importantly, COF‐TBS can be also prepared through a large‐scale synthesis. All these advantages support it as a benchmark proton conduction material.

**Figure 6:**
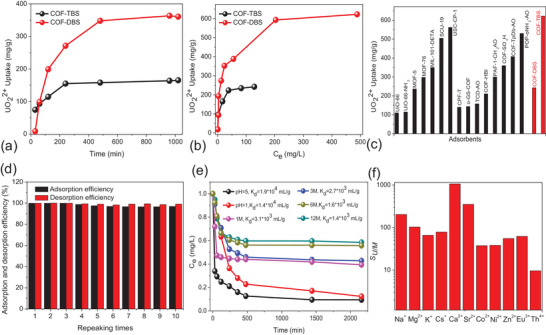
a) Adsorption kinetics of COF‐TBS and COF‐DBS at 298 K for 100 ppm UO_2_^2+^ solution. b) Adsorption isotherms of COF‐TBS and COF‐DBS at 298 K. c) A comparison of UO_2_^2+^ uptake capacity between established adsorbents and our materials of COF‐TBS and COF‐DBS. d) The reusability of COF‐TBS and COF‐DBS. e) A comparison of *K_d_* value under various acidity from a 1 ppm UO_2_^2+^ solution. f) Selectivity of UO_2_^2+^ over other metal ions from a 12 M HNO_3_ solution containing 12 mixed ions with respectively 1 ppm concentration.

The UO_2_^2+^ capture upon these COFs was initially investigated from a 100 ppm UO_2_^2+^ solution at pH = 5 (**Figure** **6**a). It was found that COF‐DBS required 8 h to reach adsorption equilibrium, which is longer than that in COF‐TBS (6 h). And these adsorption data can be well fitted by the pseudo‐second‐order models with correlation coefficient of more than 0.99 (Table S2, Supporting Information), implying chemical adsorption of UO_2_^2+^ by these HSO_3_‐pendent vinylene‐linked COFs. The adsorption capacity of 243 mg g^−1^ in COF‐TBS is below the corresponding value of 622 mg g^−1^ in COF‐DBS (Figure 6b), due to relatively low content of sulfonic units. These adsorption data was in keeping with the Langmuir model (Table S3, Supporting Information), giving theoretical adsorption capacity of 285 and 666 mg g^−1^, respectively. Remarkably, the uptake capacity in COF‐DBS exceeds the majority of reported UO_2_^2+^ materials (Figure 6c), including in polymers (POP‐oNH_2_‐AO, 530 mg g^−1^),^[^ ^16b^ ^]^ inorganic materials (FJSM‐SnS. 338 mg g^−1^),^[^ ^20^ ^]^ COFs (COF‐TpDb‐AO, 408 mg g^−1^),^[^ ^16b^ ^]^ and MOFs (USC‐CP‐1, 562 mg g^−1^, SCU‐19, 505 mg g^−1^).^[^ ^16^, ^21^ ^]^ More interestingly, COF‐DBS shows high similarity with the previously reported COF of COF‐SO_3_H^[^ ^17^ ^]^ in both structure and HSO_3_‐modification. But the only difference between them is linkage, vinylene linkage in COF‐DBS versus ketoenamine linkage in COF‐SO_3_H. However, this slight difference would lead to a significant difference in the UO_2_^2+^ uptake capacity, 622 mg g^−1^ in COF‐DBS versus 360 mg g^−1^ in COF‐SO_3_H, creating a 1.7‐fold difference, implying that enhanced stability benefits to UO_2_^2+^ capture.

To determine the affinity of COF‐DBS towards UO_2_^2+^ ions, we then tested the *K*_d_ value from a 1 ppm UO_2_^2+^ solution, giving 1.9 × 10^4^ mL g^−1^, suggesting high affinity towards UO_2_^2+^ ions, which can be ascribed to the free‐standing sulfonic units forming complexation with UO_2_^2+^ through coordination interactions. Desorption could be conveniently finished by using 3 M Na_2_CO_3_ as eluent, resulted in >99% desorption efficiency. Moreover, reusability was confirmed by repeating the adsorption‐desorption circle for ten times, where just a little decrease in UO_2_^2+^ capture was observed (Figure 6d).

Considering the excellent UO_2_^2+^ capture performance in conjunction with the strong chemical stability of COF‐DBS we further explored UO_2_^2+^ capture in rigorous condition. Generally speaking, physical adsorption would be excluded when capturing UO_2_^2+^ under strong acid, and then high affinity towards UO_2_^2+^ ions from the formation of complexation becomes the key factor to drive UO_2_^2+^ capture. For COF‐DBS, high affinity could be expected from the free‐standing sulfonic units through coordination interactions. To disclose such affinity, *K*_d_ value (mL g^−1^) was tested from a 1 ppm UO_2_^2+^ solution at various acidity, giving a hierarchy, *K*_d_^pH = 1^(1.4 × 10^4^) > *K*_d_^1M^ (3.1 × 10^3^) > *K*_d_^3M^ (2.7 × 10^3^) > *K*_d_^6M^(1.6 × 10^3^) > *K*_d_^12M^(1.4 × 10^3^) (Figure 6e), implying ultrahigh affinity even under 12 M HNO_3_. Remarkably, few adsorbents has been found to show validity under such condition of >1 M HNO_3_, due to the decomposition of materials or inactivation of functional site.^[^ ^15^ ^]^ To the best of our knowledge, this should represent the first observation of effective UO_2_^2+^ capture under 12 M HNO_3_ in the literature. The results also suggest its superior application in UO_2_^2+^ capture from the spent fuel reprocessing. Furthermore, selective adsorption of UO_2_^2+^ under 12 M HNO_3_ over a broad range of metal ions including in Na^+^, K^+^, Cs^+^, Mg^2+^, Ca^2+^, Sr^2+^, Zn^2+^, Co^2+^, Ni^2+^, Eu^3+^, Th^4+^ was observed, for example, *S_U/Ca_* = 1062, *S_U/Cs_* = 78, *S_U/Sr_* = 350, *S_U/Eu_* = 62, *S_U/Th_* = 9.6 (Figure 6f), confirming its practical application in the spent fuel reprocessing. Moreover, the inclusion of uranium on COF‐DBS can be directly read out from characterizations of XPS, EDS with elemental distribution mapping, and IR. In the XPS spectra, both U_4f_ and N_1s_ signals at 382 and 400 eV was observed in UO_2_^2+^‐loaded COF‐DBS (Figure S7, Supporting Information), suggesting the capture of UO_2_(NO_3_)_2_, since formation of neutral species UO_2_(NO_3_)_2_ will occur under 12 M HNO_3_. Electron transfer leading to decrease in the electron binding energy of S_2p_ ≈0.4 eV was observed for the UO_2_^2+^‐loaded samples (Figure S8, Supporting Information), relative to pristine samples, owing to the coordination interactions between UO_2_(NO_3_)_2_ and sulfonic units. In the characterization of EDS with elemental distribution mapping, homogeneous distribution of uranium and nitrogen elements was observed (Figure S9, Supporting Information), confirming the UO_2_(NO_3_)_2_ capture. In IR spectra, new peak at 922 cm^−1^ was observed (Figure S10, Supporting Information), which was assigned to the antisymmetric vibration of uranyl ions. This, relative to UO_2_(NO_3_)_2_·6H_2_O with peak at 960 cm^−1^,^[^ ^17^ ^]^ showed big redshift, owing to strong coordination interactions between sulfonic units and uranyl ions.

**Figure 7:**
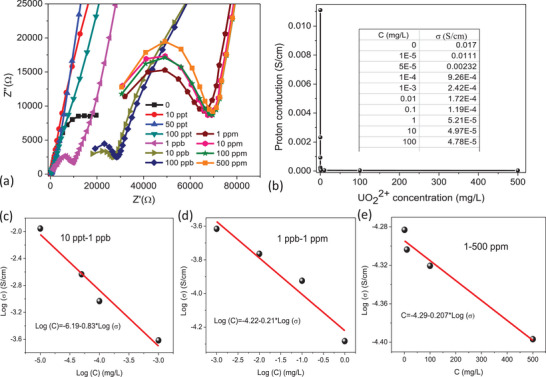
a) Proton‐conduction sensing of UO_2_^2+^ ions at 0–500 ppm upon COF‐TBS. b) A comparison of proton conductivity at 0–500 ppm. c) The linear relationship at 10 ppt‐1 ppb. d) The linear relationship at 1 ppb‐1 ppm. e) The linear relationship at 1–500 ppm.

The excellent proton conduction performance in conjunction with the exceptional chemical and shape stability in COF‐TBS urged us to further explore UO_2_^2+^ sensing upon proton conductivity. First, the response time was tested at 298 K through soaking one COF‐TBS block sample in 1 ppb UO_2_^2+^ solution with pH = 5 for 0–4 min, and fast response within 2 min was found to be the optimal response time (Figure S11, Table S4, Supporting Information). Then, UO_2_^2+^ sensing was investigated through soaking one COF‐TBS block sample in 10 ppt‐500 ppm UO_2_^2+^ solution with pH = 5 for 2 min and the results were shown in **Figure** **7**a,b. The proton conductivity after soaking in 10 ppt UO_2_^2+^ solution was 1.11 × 10^−2^ S cm^−1^, lower than that in pristine sample of COF‐TBS (1.7 × 10^−2^ S cm^−1^), suggesting smart proton‐conduction response towards UO_2_^2+^ ions. Interestingly, this proton‐conductivity sensing was composed of three sections, viz. 10 ppt‐1 ppb, 1 ppb‐1 ppm, and 1–500 ppm. For 10 ppt‐1 ppb section, a Log (C) versus Log (*σ*) plot gave a linear relationship of Log (C) = −6.19‐0.83 × Log (*σ*) with *R* = 0.96 (Figure 7c), where *C* and *σ* was the concentration of UO_2_^2+^ solution and the proton conductivity for the sample after soaking corresponding UO_2_^2+^ solution, respectively. Similarly, linear relationship of Log (C) = −4.22–0.21 × Log (*σ*) with *R* = 0.92 was observed in 1 ppb‐1 ppm (Figure 7d), while 1–500 ppm gave a linear relationship of C = −4.29‐0.207 × Log (*σ*) with *R* = 0.96 (Figure 7e). The results suggest the superior application of COF‐TBS for the determination of UO_2_^2+^ concentration through solid‐state proton‐conduction technique with fast response time of 2 min and ultralow detection concentration of 10 ppt. As we know, the concentration of UO_2_^2+^ was generally determined by UV‐vis spectrometer and ICP‐OES with the detection concentration down to ppm level. To determine lower concentration such as ppb level, commercial ICP‐MS was employed. However, there is still a lack of effective techniques to determine the concentration of ppt level up to date. Maybe, our solid‐state proton‐conduction technique would be a promising solution. Moreover, this technique also afforded extremely large determination range from ppt to ppm with six orders of magnitude span, which is never observed for all established technique for UO_2_^2+^ detection. This outstanding merit is mainly due to the excellent proton conduction ability and smart response towards UO_2_^2+^ ions derived from the pendent –HSO_3_ units that afford strong affinity towards UO_2_^2+^ ions through coordination interactions. To confirm its practical application, ion interference involving in 11 other ions solution with concentration ratio of C_U_/C_M_ = 1:1, 1:10, and 1:50(C_U_ = 1 ppb) was tested (Figure S12, Table S5, Supporting Information), and almost no detectable change was observed, relative to the value from just 1 ppb UO_2_^2+^, excluding ion interference. Moreover, to disclose the affinity of COF‐TBS towards UO_2_^2+^ ions, *K*_d_ value was tested from a 1 ppm UO_2_^2+^ solution, giving *K*_d_ = 6.9 × 10^4^ mL g^−1^ (Figure S13, Supporting Information), suggesting big affinity from the free‐standing ‐SO_3_H units.

## 3 Conclusion

In summary, we demonstrate a proof‐of‐concept of synthesis of vinylene‐linked COFs via using HSO_3_‐activated approach. As a result, two HSO_3_‐pendent vinylene‐linked COFs were constructed through a melt polymerization approach with gram scale. Stability tests disclosed ultrahigh stability of these two COFs even under some extreme conditions. Proton conduction tests showed outstanding performance for COF‐TBS with ultrahigh proton conductivity value up to 7.28×10^−2^ S cm^−1^ at 358 K, ultralow activation energy down to 0.086 eV, and excellent durability extended to 15 days. UO_2_^2+^ uptake tests enabled unique affinity and validity towards UO_2_^2+^ ions under 12 M HNO_3_ for COF‐DBS. More impressively, the combination of proton conduction and UO_2_^2+^ uptake, for the first time, brings a promising proton‐conductivity sensing of UO_2_^2+^ ions for detection of UO_2_^2+^ ions with ultralow detection concentration down to 10 ppt and broad detection scope up to six orders of magnitude. All these outstanding results are based on the key factor of HSO_3_‐pendent vinylene‐linked COF, while all these exciting results strongly suggest its superior potential in fuel cells, spent fuel reprocessing, and detection of UO_2_^2+^ ions.

## Conflict of Interest

The authors declare no conflict of interest.

## Supporting information

Supporting Information

## Data Availability

The data that support the findings of this study are available in the supplementary material of this article.

## References

[1] a) K. D. Kreuer , S. J. Paddison , E. Spohr , M. Schuster , Chem. Rev. 2004, 104, 4637; 15669165 10.1021/cr020715f

[2] a) X. Meng , H. N. Wang , S. Y. Song , H. J. Zhang , Chem. Soc. Rev. 2017, 46, 464; 27918056 10.1039/c6cs00528d

[3] a) R. Sahoo , S. Mondal , S. C. Pal , D. Mukherjee , M. C. Das , Adv. Energy Mater. 2021, 11, 2102300;

[4] a) K. A. Mauritz , R. B. Moore , Chem. Rev. 2004, 104, 4535; 15669162 10.1021/cr0207123

[5] a) L. S. Xie , G. Skorupskii , M. Dincă , Chem. Rev. 2020, 120, 8536; 32275412 10.1021/acs.chemrev.9b00766 PMC7453401

[6] a) B. Wang , R.‐B. Lin , Z. J. Zhang , S. C. Xiang , B. L. Chen , J. Am. Chem. Soc. 2020, 142, 14399; 32786796 10.1021/jacs.0c06473

[7] P. Li , J. Chen , S. Tang , Chem. Eng. J. 2021, 415, 129021.

[8] S. Tao , L. Zhai , A. D. D. Wonanke , M. A. Addicoat , Q. Jiang , D. Jiang , Nat. Commun. 2020, 11, 1981. 32332734 10.1038/s41467-020-15918-1 PMC7181855

[9] Z. Wang , Y. Yang , Z. Zhao , P. Zhang , Y. Zhang , J. Liu , S. Ma , P. Cheng , Y. Chen , Z. Zhang , Nat. Commun. 2021, 12, 1982. 33790298 10.1038/s41467-021-22288-9 PMC8012354

[10] Y. Peng , G. Xu , Z. Hu , Y. Cheng , C. Chi , D. Yuan , H. Cheng , D. Zhao , ACS Appl. Mater. Interfaces 2016, 8, 18505. 27385672 10.1021/acsami.6b06189

[11] F. Yang , G. Xu , Y. Dou , B. Wang , H. Zhang , H. Wu , W. Zhou , J. Li , B. Chen , Nat. Energy 2017, 2, 877.

[12] J. Chen , Q. Mei , Y. Chen , C. Marsh , B. An , X. Han , I. P. Silverwood , M. Li , Y. Cheng , M. He , X. Chen , W. Li , M. Kippax‐Jones , D. Crawshaw , M. D. Frogley , S. J. Day , V. García‐Sakai , P. Manuel , A. J. Ramirez‐Cuesta , S. Yang , M. Schröder , J. Am. Chem. Soc. 2022, 144, 11969. 35775201 10.1021/jacs.2c04900 PMC9348827

[13] a) C. Tsouris , Nat. Energy 2017, 2, 17022;

[14] a) M. Gavrilescu , L. V. Pavel , I. Cretescu , J. Hazard. Mater. 2009, 163, 475; 18771850 10.1016/j.jhazmat.2008.07.103

[15] a) M. Xu , X. Han , T. Wang , S. Lia , D. Hua , J. Mater. Chem. A 2018, 6, 13894;

[16] a) H. Zhang , W. Liu , A. Li , D. Zhang , X. Li , F. Zhai , L. Chen , L. Chen , Y. Wang , S. Wang , Angew. Chem., Int. Ed. 2019, 58, 16110; 10.1002/anie.201909718 31518048

[17] a) X. H. Xiong , Z. W. Yu , L. L. Gong , Y. Tao , Z. Gao , L. Wang , W. H. Yin , L. X. Yang , F. Luo , Adv. Sci. 2019, 6, 1900547; 10.1002/advs.201900547 PMC6702651 31453066

[18] a) H. Li , F. Chen , X. Guan , J. Li , C. Li , B. Tang , V. Valtchev , Y. Yan , S. Qiu , Q. Fang , J. Am. Chem. Soc. 2021, 143, 2654; 33567211 10.1021/jacs.0c12499

[19] a) F. Meng , S. Bi , Z. Sun , D. Wu , F. Zhang , Angew. Chem., Int. Ed. 2022, 61, e202210447; 10.1002/anie.202210447 36099563

[20] M. L. Feng , D. Sarma , X. H. Qi , K. Z. Du , X. Y. Huang , M. G. Kanatzidis , J. Am. Chem. Soc. 2016, 138, 12578. 27584863 10.1021/jacs.6b07351

[21] X. Wang , Y. Chen , L. Song , Z. Fang , J. Zhang , F. Shi , Y. Lin , Y. Sun , Y. Zhang , J. Rocha , Angew. Chem., Int. Ed. 2019, 58, 18808. 10.1002/anie.201909045 31609512

