# Supplementary material for: Sulfonic‐Pendent Vinylene‐Linked Covalent Organic Frameworks Enabling Benchmark Potential in Advanced Energy
Source: Adv Sci (Weinh). 2023 Mar 1;10(13):2300408. doi: 10.1002/advs.202300408 (PMC10161031; doi:10.1002/advs.202300408)
Supplement: Supplementary file 1 — Supporting Information [file ADVS-10-2300408-s001.pdf]

Supporting Information

Sulfonic-Pendent Vinylene-Linked Covalent Organic Frameworks Enabling Benchmark Potential in Advanced Energy

*Ying Xu,[a] Zhiwu Yu,[b] Qingyun Zhang,[a] and Feng Luo[a]**

1. Materials and general methods

Reagents and solvents were commercially available (Alfa) and were used without further purification. X-ray powder diffraction were collected by a Bruker AXSD8 Discover powder diffractometer at 40 kV, 40 mA for Cu Kλ (λ= 1.5406 Å). The simulated powder patterns were calculated by Mercury 1.4. Infrared Spectra (IR) were measured by a Bruker VERTEX70 spectrometer in the 500-4000 cm^{-1} region. The gas adsorption isotherms were collected on a Belsorp-max. Ultrahigh-purity-grade (>99.999%) N_2 gases were used during the adsorption measurement. SEM and EDS measurements were carried out using a Hitachi S-4800 microscope. The analyses of concentrations of metal ions in the solution was carried out by ThermoFisher iCap7600 ICP-OES instruments. X-ray photoelectron spectra (XPS) were collected by Thermo Scientific ESCALAB 250 Xi spectrometer. Solid-state NMR experiments were performed on Varian Infinityplus 300 solid-state NMR spectrometer (300MHz).

2. Impedance measurements

Proton conductivity of the COFs was measured by AC impedance using Ivium CompactStat potentiostat B31250 under controlled humidity and temperature. About 10 mg materials were cutted into quadrate pellets (4.7×2.3 mm in diameter, 1 mm in thickness) from the big block samples. The quadrate pellets were placed between two-electrode cell connected with Ivium CompactStat potentiostat B31250 by a conductive wire. The temperature dependence of proton conductivity was tested by EIS with a tuned frequency range from 1 Hz to 1MHz and an alternating potential of 100 mV in a humidity chamber maintained at 98% RH. The humidity dependence of proton conductivity was determined using different humidities controlled by saturated salt aqueous solutions in a constant temperature and humidity chamber. When changing temperature or humidity, the pellets were equilibrated for 6 h. Proton conductivity (σ, S cm^{-1}) was calculated by the following equation:

$$\sigma = L/RA \qquad (1)$$

where σ is the proton conductivity (S cm^{-1}), L is the thickness of the pellet (cm), A is the area of the pellet (cm^2), and R is the resistance (Ω) of the pellet corresponding to the real Z′ Nyquist plot.

Activation energy (E_a) of proton conductivity (σ) was extracted from the data measured at various temperatures (98% RH) by using the Arrhenius equation:

$$\sigma = (\sigma_0/T)\cdot \exp[-E_a/(k\cdot T)] \qquad (2)$$

where σ_0, T and k are the pre-exponential factor, temperature and Boltzmann constant, respectively.

3. Synthesis

3.1 Synthesis of model compound of 2,4,6-tri(styryl)benzenesulfonic acid

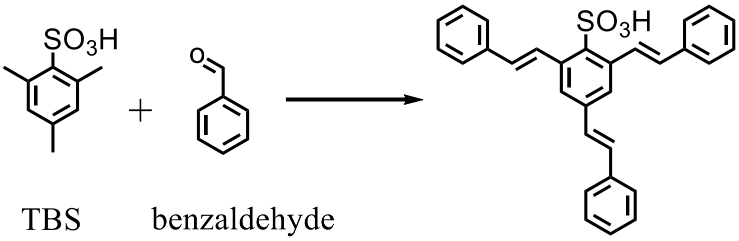

TBS benzaldehyde

Model compound of 2,4,6-tri(styryl)benzenesulfonic acid was prepared as follows. A mixture of TBS (0.1 mmol), benzaldehyde (0.3 mmol), and benzoic anhydride (0.3 mmol) was mixed in the sealed tube and heated at 200°C for three days, then cooled to room temperature. The products were recrystallization from water to give the pure product as a colorless powder. Mass spectrometry (HR-MS) was used to confirm the product and purity. EA (%): calc. C/77.55, H/5.21; exp. C/77.21, H/5.16.

3.2 Synthesis of model compound of 2,5-bis(-2-hydroxystyryl)benzenesulfonic acid

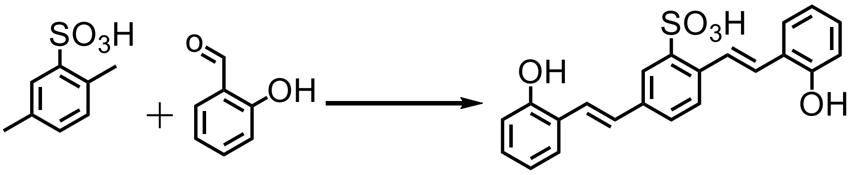

DBS 2-hydroxybenzaldehyde

Model compound of 2,5-bis(-2-hydroxystyryl)benzenesulfonic acid was prepared as follows. A mixture of DBS (0.1 mmol), 2-hydroxybenzaldehyde (0.2 mmol), and benzoic anhydride (0.3 mmol) was mixed in the sealed tube and heated at 200°C for three days, then cooled to room temperature. The products were recrystallization from water to give the pure product as a colorless powder. Mass spectrometry (HR-MS) was used to confirm the product and purity. EA (%): calc. C/67.51, H/3.86; exp. C/67.12, H/3.78.

3.3 Synthesis of COF-TBS

COF-TBS was prepared as follow. A mixture of TBS (400 mg, 2 mmol), 2,5-divinylterephthalaldehyde (552 mg, 3 mmol), and benzoic anhydride (676 g, 3 mmol) was mixed in the sealed tube and heated at 200°C for seven days, then cooled to room temperature. The solid was collected and washed with DMF and methanol. The block was

dried at 100 °C under vacuum overnight to give the expected COFs in ~94% isolated yield. EA (%): calc. C/76.75, H/4.29; exp. C/76.26, H/4.

3.4 Synthesis of COF-DBS

COF-DBS was prepared as follow. A mixture of DBS (372 mg, 2 mmol), 2,4,6-trihydroxybenzene-1,3,5-tricarbaldehyde (630 mg, 3 mmol), and benzoic anhydride (676 mg, 3 mmol) was mixed in the sealed tube and heated at 200°C for seven days, then cooled to room temperature. The solid was collected and washed with DMF and methanol. The block was dried at 100 °C under vacuum overnight to give the expected COFs in ~96% isolated yield. EA (%): calc. C/57.92, H/3.47; exp. C/57.88, H/3.34.

4. Stability tests

100 mg samples of these COFs were soaked in 30 mL 12M HNO_3 solution for three days. Then IR, PXRD, CPMAS-ssNMR, and N_2 adsorption was employed to characterize the samples after filtration. Similarly, IR, PXRD, CPMAS-ssNMR, and N_2 adsorption was employed to characterize the samples after β-irradiation with 200 kGy dose.

5. Uranium uptake *via* batch experiments

U(VI) solution was prepared by dissolving uranyl nitrate ($UO_2(NO_3)_2{\cdot}6H_2O$, analytical reagent) in deionized water. The pH value is adjusted by HNO_3 (1 M). Adsorption temperature is 298 K.

In kinetics experiments, the U solution with initial concentration of 100 ppm and pH=5 was used. The dose of adsorbent is 10 mg, while the U solution is 20 mL.

In isotherm experiments, the U solution with initial concentration of 10-800 ppm and pH=5 was used. The dose of adsorbent is 10 mg, while the U solution is 20 mL and the contact time is 8 h.

In determining affinity experiments, the U solution with initial concentration of 1 ppm and pH=5, 1 and 1-12 M HNO_3 was used. The dose of adsorbent is 10 mg, while the U solution is 20 mL.

In selective adsorption experiments, a 12-ions mixed solution (12M HNO_3) contains both U and other 11 metal ions with respectively initial concentration of 1 ppm was used. The dose of adsorbent is 10 mg, while the solution is 20 mL.

6. Some related calculation and fitting in this U adsorption experiments

The adsorption amount, Q_e (mg/g), was calculated by the difference of the U(VI) equilibrium concentration before and after adsorption (see equation 3):

$$Q_e = \frac{(C_0 - C_e) \times V}{m} \qquad (3)$$

where c_0 (mg/L) and c_e (mg/L) are the initial concentration and equilibrium concentration of uranium in the solutions, respectively; V (mL) is the volume of testing solution and m (mg) is the amount of sorbent.

The adsorption kinetics was analyzed by simplified kinetic models such as the pseudo-first-order and pseudo-second-order, through the following two equations,

$$\ln(Q_e - Q_t) = \ln Q_e - k_1 \qquad (4)$$

$$\frac{t}{Q_t} = \frac{1}{k_2 \times {q_e}^2} + \frac{t}{Q_e} \qquad (5)$$

Where Qe (mg/g) and Qt (mg/g) are the quantity of the adsorbed U(VI) at equilibrium and at t time, respectively, and K_1 (min^{-1})/K_2[g/(mg•min)] is the pseudo-first/second-order sorption rate constant that is deduced from the slope of the plot of t/Qt versus t.

The isotherm data was fitted by Langmuir and Freundlich models *via* the following two equations,

$$\frac{c_e}{Q_e} = \frac{c_e}{Q_m} + \frac{1}{k_L Q_m} \qquad (6)$$

$$\ln Q_e = \ln k_F + \frac{1}{n} \ln c_e \qquad (7)$$

Where Q_e (mg/g) is the amount adsorbed at equilibrium and C_e (mg/L) is the equilibrium concentration; Q_m(mg/g) is the maximum adsorption amount; K_L (L/mg) is an equilibrium constant related to the binding strength; n and K_F (L/mg) are Freundlich constants which are indicators of the adsorption capacity and adsorption intensity, respectively.

The K_d value and selectivity (S) is calculated from the following two equations,

$$K_d = \frac{V}{m} \frac{(C_0 - C_e)}{C_e} \times 10^3 \qquad (8)$$

$$S = \frac{K_d^U}{K_d^M} \qquad (9)$$

where the unit for K_d value is mL/g.

7. Proton-conductivity Sensing of UO_2^{2+}

The response time was tested follows. Quadrate pellets of COF-TBS were soaked in 1 ppb UO_2^{2+} solution with pH=5 for 0, 0.5 min, 1 min, 2 min, 3 min, 4 min, respectively, then the quadrate pellets were taken out of the solution and dried naturally for six hours. Next, the proton conductivity of these resultant quadrate pellets was analyzed by AC impedance spectroscopy at 298 K under RH= 98%.

Sensing at various UO_2^{2+} concentration was tested follows. Quadrate pellets of COF-TBS were soaked in various UO_2^{2+} solutions with pH=5 for 2 min, then the quadrate pellets were taken out of the solution and dried naturally for six hours. Next, the proton conductivity of

these resultant quadrate pellets was analyzed by AC impedance spectroscopy at 298 K under RH= 98%.

Ion interference was tested follows. Quadrate pellets of COF-TBS were soaked in a 12-ions mixed solution containing both U and other 11 metal ions with respectively initial concentration of 1 ppm and pH=5 for 2 min, then the quadrate pellets were taken out of the solution and dried naturally for six hours. Next, the proton conductivity of these resultant quadrate pellets was analyzed by AC impedance spectroscopy at 298 K under RH= 98%.

8. Additional Figures and Tables

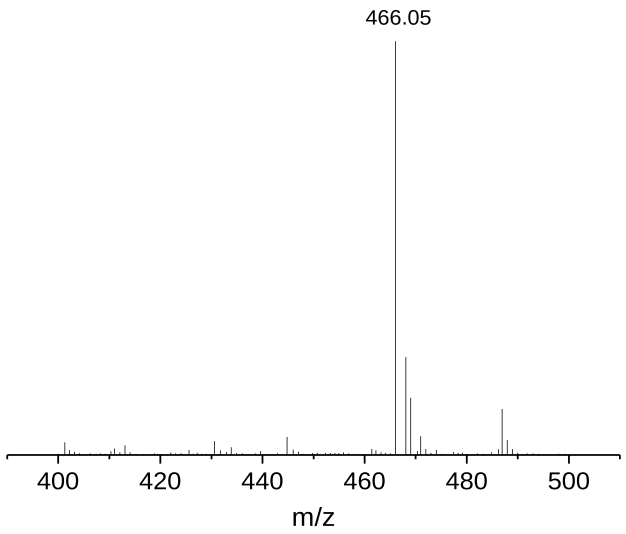

Figure S1. The mass spectrometry (HR-MS) of 2,5-bis(-2-hydroxystyryl)benzenesulfonic acid.

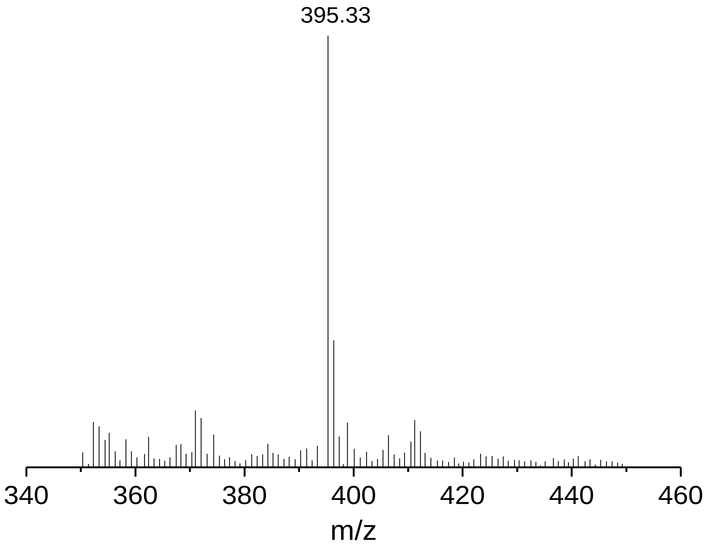

Figure S2. The mass spectrometry (HR-MS) of 2,4,6-tri(styryl)benzenesulfonic acid.

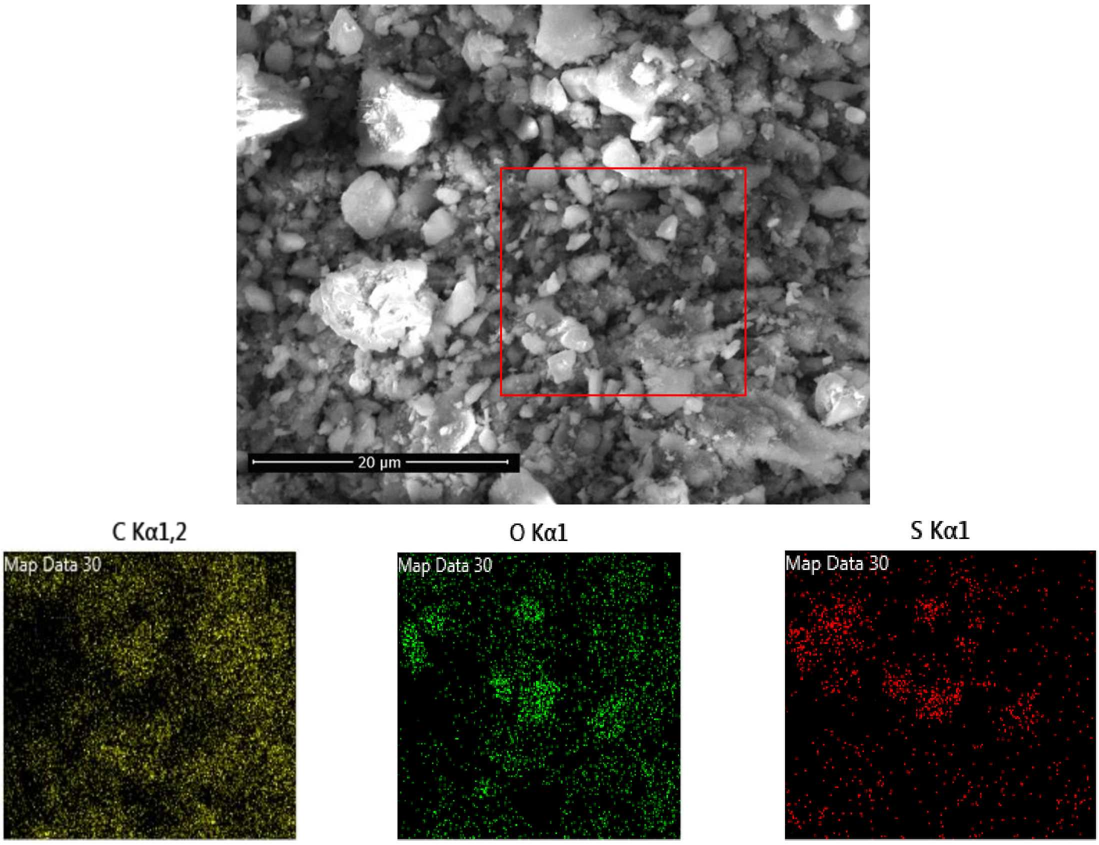

Figure S3. The SEM image and EDS element mapping for COF-TBS.

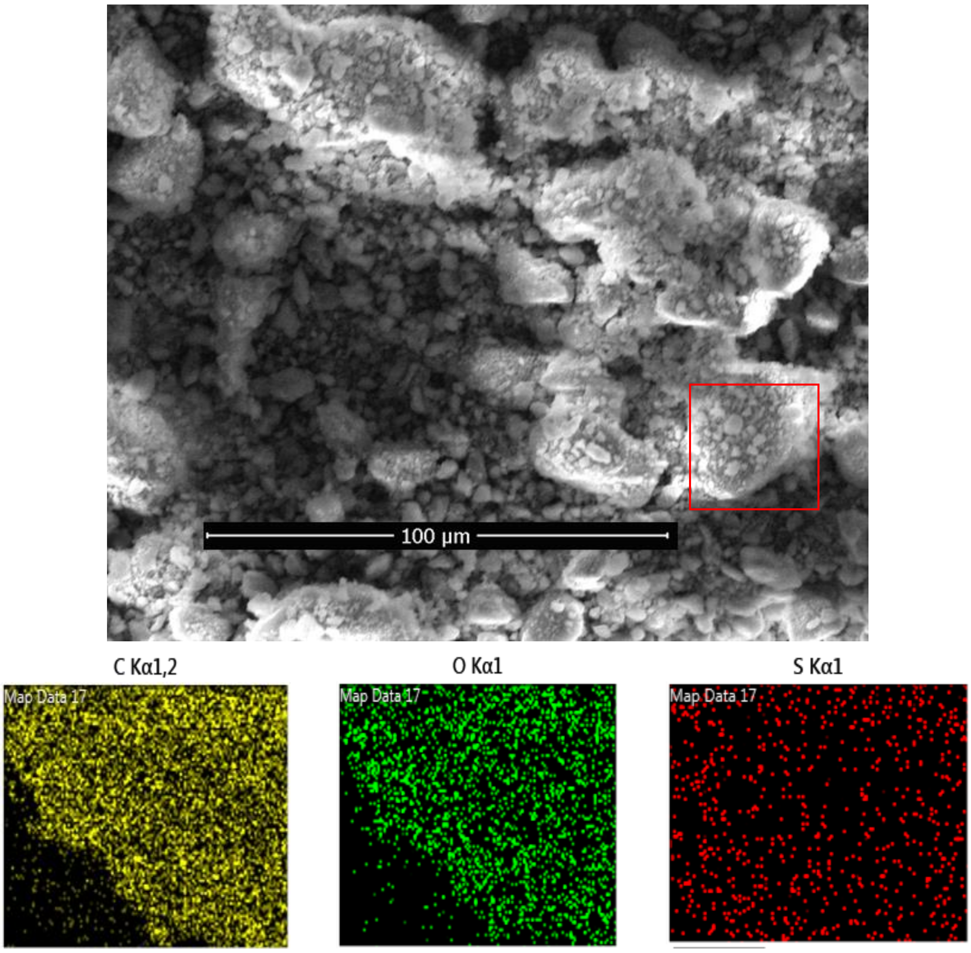

Figure S4. The SEM image and EDS element mapping for COF-DBS.

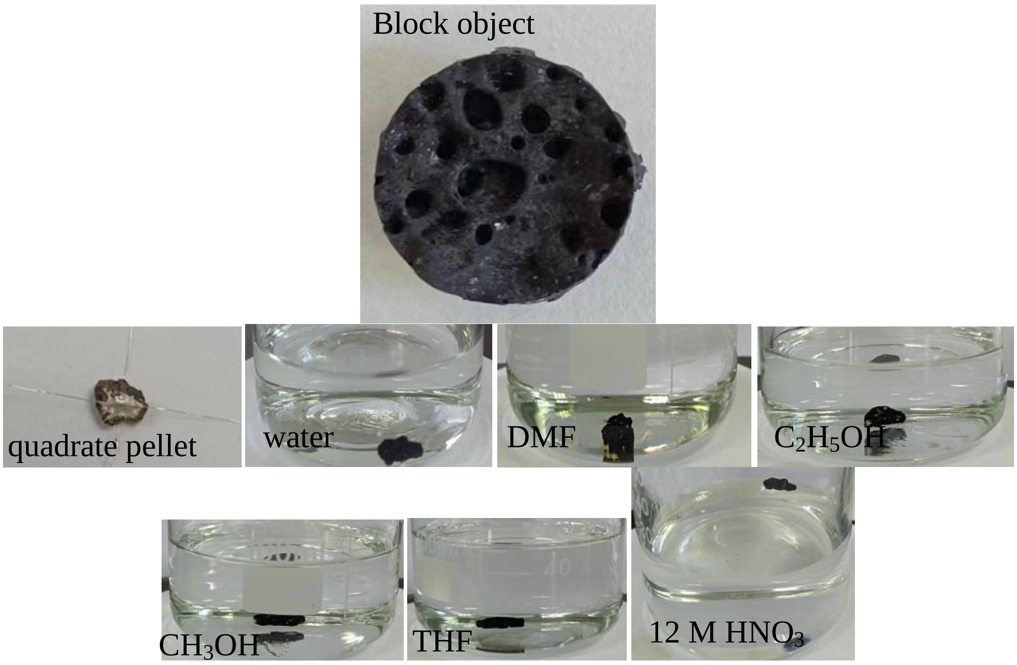

Figure S5. The images of the block object from this melt polymerization synthesis, the cutted quadrate pellet for proton conduction determination, and the cutted pellets after immersing in vairous solvents for one week.

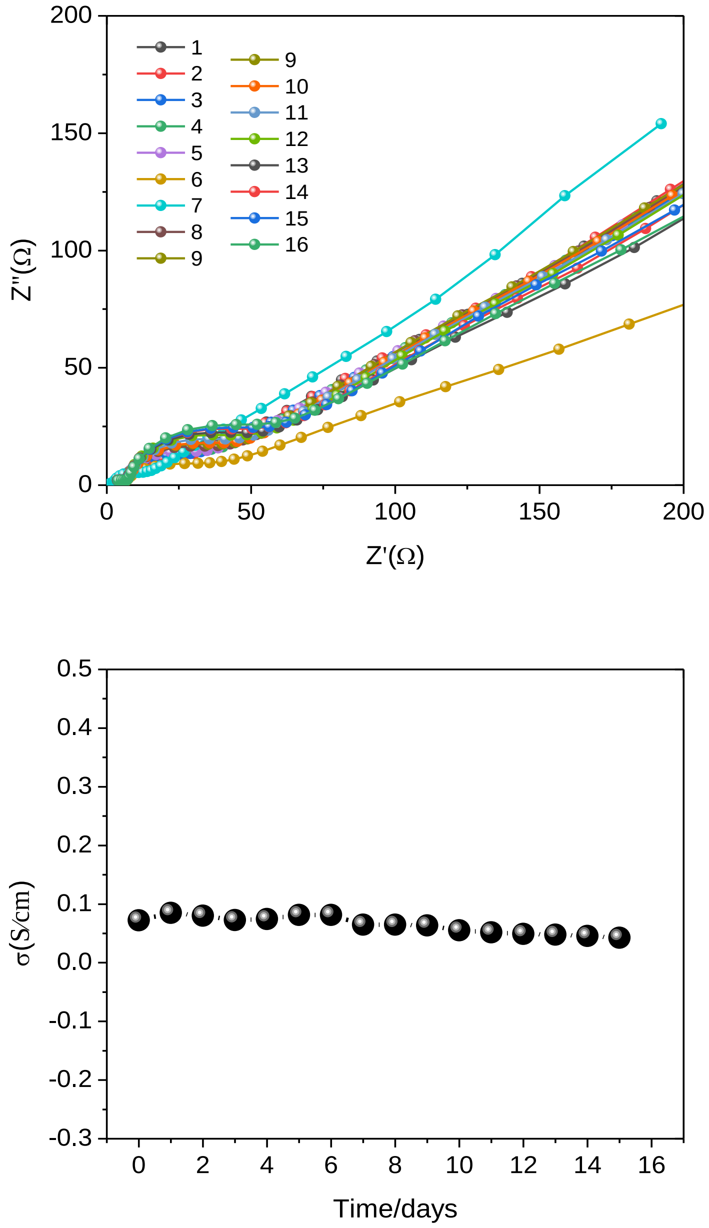

Figure S6. Durability tests of COF-TBS at 358 K and RH=98% for 15 days.

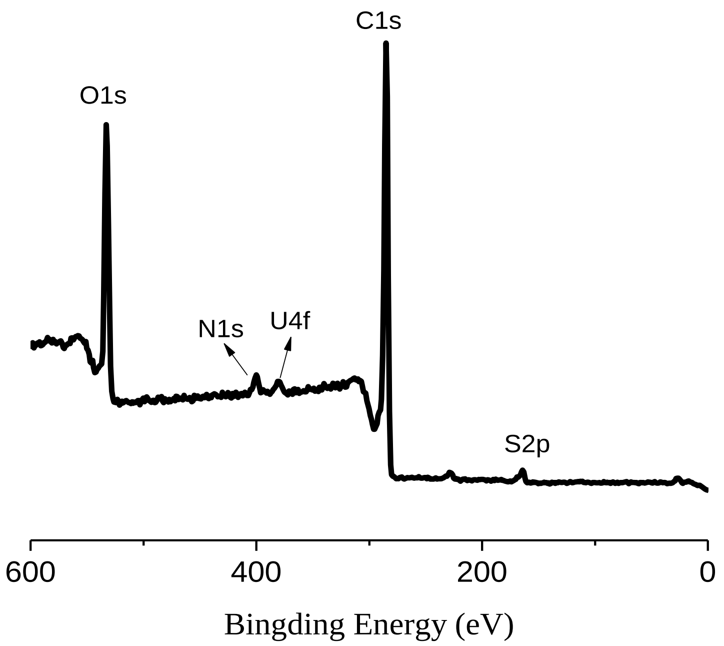

Figure S7. XPS spectrum of COF-DBS after adsorption of UO_2^{2+} from 1 ppm UO_2^{2+} solution (12M HNO_3).

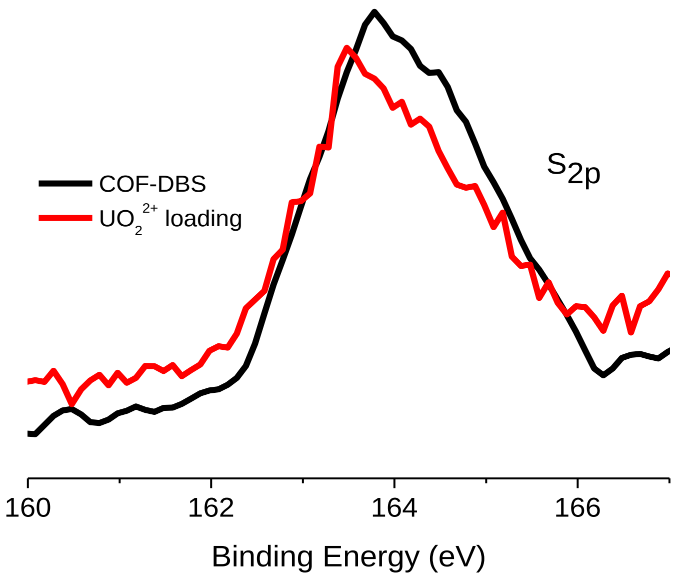

Figure S8. A comparison of HRXPS of S_{2p} between as-synthesized samples and samples after adsorption of UO_2^{2+} from 1 ppm UO_2^{2+} solution (12M HNO_3).

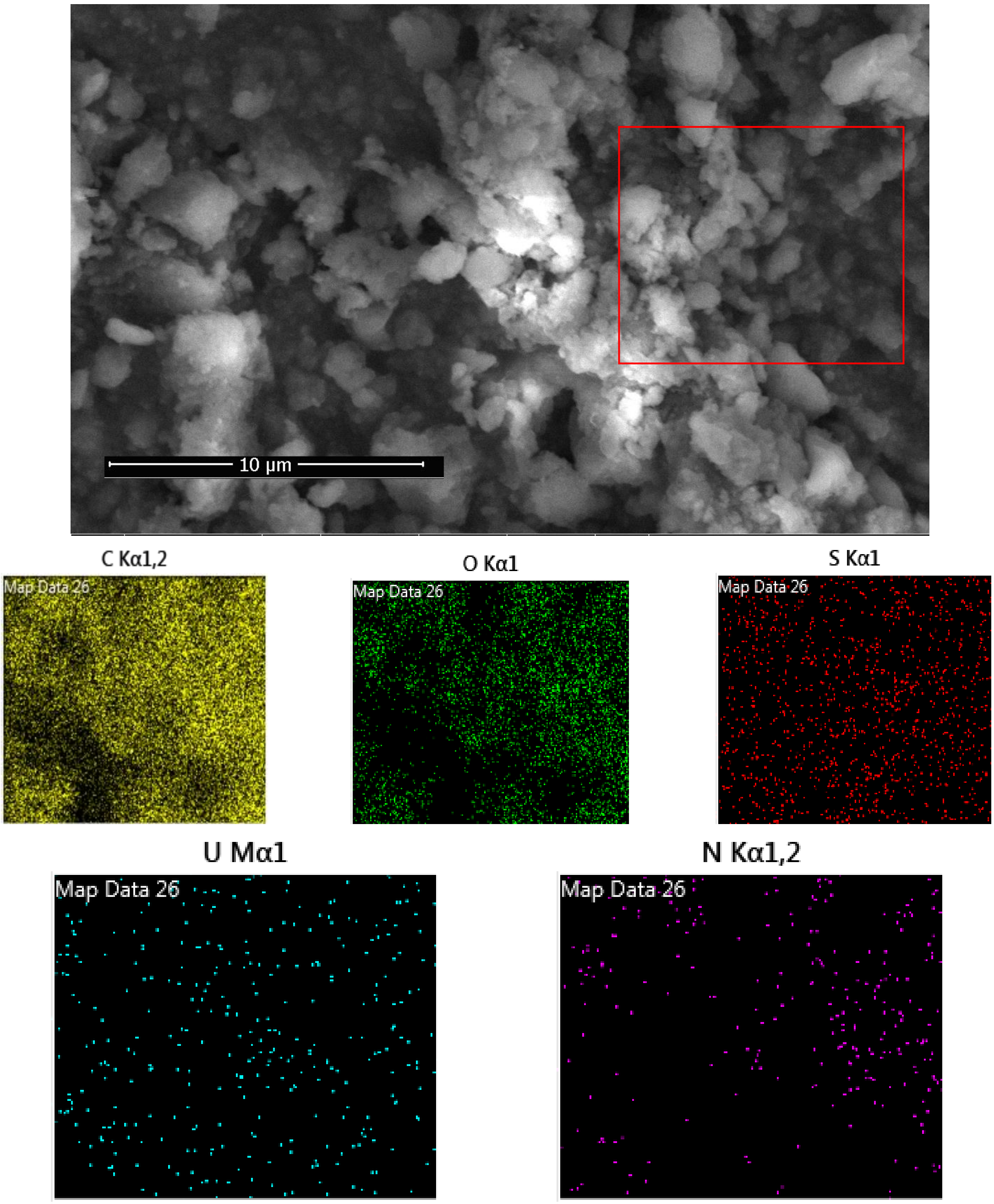

Figure S9. The SEM image and EDS element mapping for COF-DBS after adsorption of UO_2^{2+} from 1 ppm UO_2^{2+} solution (12M HNO_3).

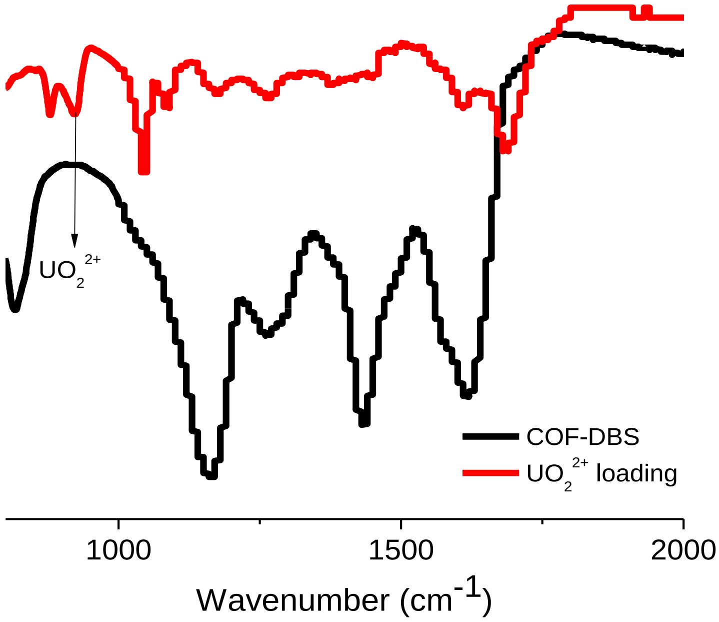

Figure S10. A comparision of IR between as-synthesized samples and samples after adsorption of UO_2^{2+} from 1 ppm UO_2^{2+} solution (12M HNO_3).

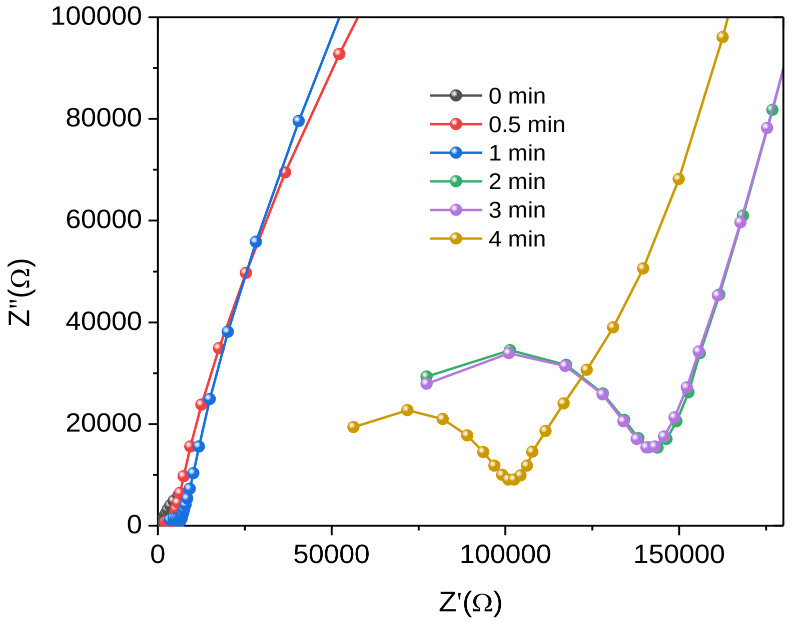

Figure S11. Time response of proton-conduction sensing upon COF-DBS.

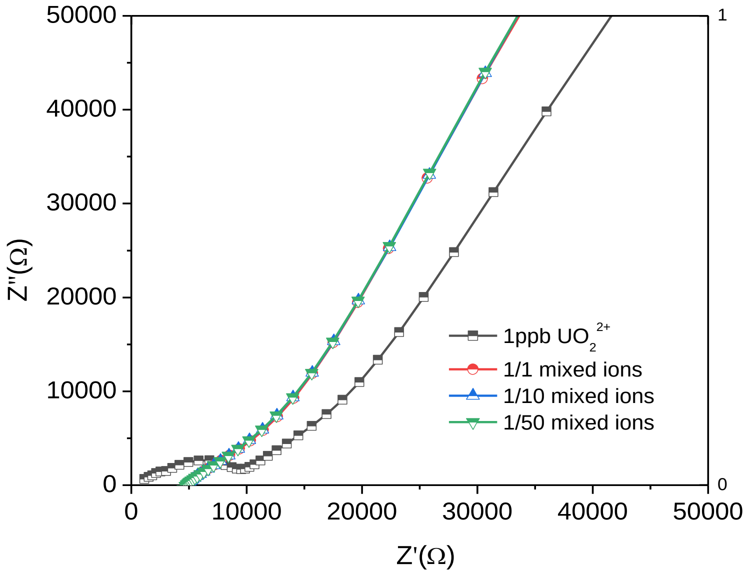

Figure S12. Ion interference of this proton-conductivity sensing under 12-ions mixed solution with concentration ratio of C_U/C_M=1:1, 1:10, and 1:50 (C_U=1 ppb).

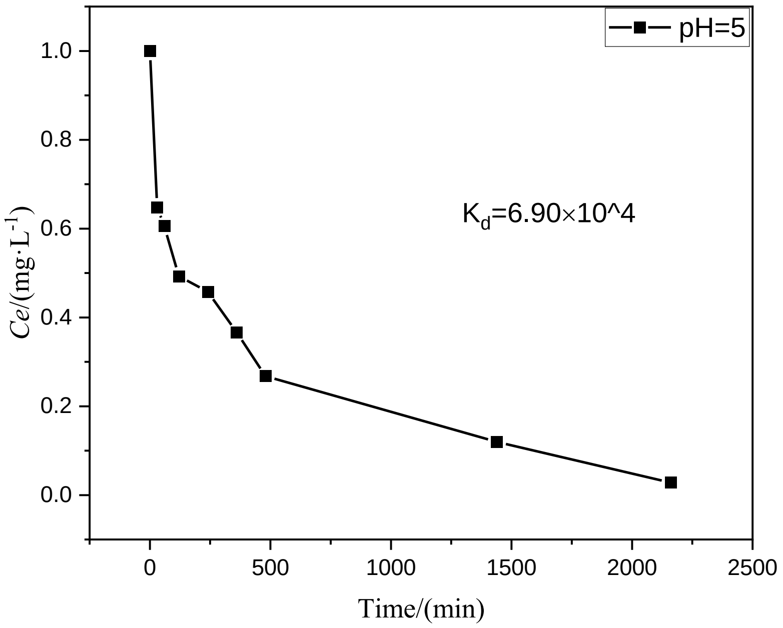

Figure S13. K_d value of COF-TBS.

Table S1. List of the established outstanding proton conductive materials.

Materials	σ (S/cm)	RH (%)	T (K)	E_a (eV)	Durability (hours)	Source	References
Nafion	0.1	98%	353	0.22	>360	intrinsic	J. Polym. Sci., Part B: Polym. Phys. 2011, 49, 1437
H_3PO_4@NKCOF-10	0.0697	90	298	0.06	48	extrinsic	Nat. Commun. 2021, 12, 1982
H_3PO_4@TPB-DMeTP-COF	0.19	0	433	0.34	24	extrinsic	Nat. Commun. 2020, 11, 1981
COF-F6-H	0.042	0	413	-	45	intrinsic	J. Am. Chem. Soc, 2020, 142, 14357
BIP(COF)	0.032	95	368	0.31	15	intrinsic	J. Am. Chem. Soc. 2019, 141, 14950
im@TPB-DMTP-COF	0.00437	0	403	0.38	24	extrinsic	Nat. Mater. 2016, 15, 722
Il-COF-SO_3H@SNF-35	0.224	100	90	0.08	60	extrinsic intrinsic	Adv. Energy Mater. 2021, 11, 2102300
SPEEK/HPWQ@COF	0.08	100	75	0.24	360	extrinsic	Nat. Rev. Mater. 2019, 4, 708
NUS-10(R)	0.04	97	25	0.2	360	intrinsic	ACS Appl. Mater. Interfaces 2016, 8, 18505
BUT-8(Cr)A	0.076	100	25	0.11	40	intrinsic	Nat. Energy 2017, 2, 877-883
MFM-300(Cr)·$SO_4(H_3O)_2$	0.0126	99	25	0.04	>360	extrinsic	J. Am. Chem. Soc. 2022, 144, 11969-11974
COF-DBS	0.00224	98	80	0.15	-	intrinsic	**This work**
COF-TBS	0.0728	98	80	0.086	360	intrinsic	**This work**

'-' denotes no data that can be obtained.

Table S2. Fitting these data by the pseudo-first-order and pseudo-second-order models.

Adsorbents	$q_{e,\mathrm{exp}}$ (mg·g^{-1})	pseudo-first-order			pseudo-second-order		
		$q_{e,\mathrm{cal}}$ (mg·g^{-1})	k_1 (min^{-1})	R^2	$q_{e,\mathrm{cal}}$ (mg·g^{-1})	k_2 (g·mg^{-1}·min^{-1})	R^2
COF-DBS	195.135	139.156	0.0658	0.966	195.069	0.000394	0.9978
COF-TBS	165.217	70.485	0.0099029	0.9069	163.256	0.00014155	0.9994

Table S3. Fitting these data by the Langmuir and Freundlich models.

Adsorbents	Langmuir model				Freundlich model		
	q_e, exp (mg·g-1)	K_L (L·mg-1)	q_m (mg·g-1)	R^2	K_F (mg1-1/n·g-1·L-1/n)	1/n	R^2
COF-DBS	622	0.05281	666.667	0.9983	64.06512	0.4318	0.9349
COF-TBS	237.174	0.217391304	250	0.9988	6035.4156	2.3173	0.9094

Table S4. Time respose towards 1ppb UO_2^{2+} solution.

Sample	Temperature(K)	Time (min)	Proton conductivity (S cm^{-1})
COF-TBS	298K	0	1.75×10^{-2}
		0.5	8.58×10^{-4}
		1	5.36×10^{-4}
		2	2.54×10^{-4}
		3	2.66×10^{-4}
		4	3.77×10^{-4}

Table S5. Ion interference involving in 11 other ions solution with concentration ratio of C_U/C_M= 1:1, 1:10, and 1:50 (C_U=1 ppb).

Sample	Temperature(K)	Ion concentration	Proton conductivity (S cm^{-1})
COF-TBS	298K	1ppb	2.42×10^{-4}
		1/1	2.44×10^{-4}
		1/10	2.44×10^{-4}
		1/50	2.36×10^{-4}
